# MicroRNA‐34a in coronary heart disease: Correlation with disease risk, blood lipid, stenosis degree, inflammatory cytokines, and cell adhesion molecules

**DOI:** 10.1002/jcla.24138

**Published:** 2021-12-03

**Authors:** Hefei Li, Mingchao Chen, Qiang Feng, Lin Zhu, Zhichao Bai, Boya Wang, Zhangli Guo, Aijun Hou, Hui Li

**Affiliations:** ^1^ Department of Cardiology HanDan Central Hospital Handan China; ^2^ Intensive Care Unit Department Affiliated Hospital of Hebei University of Engineering Handan China

**Keywords:** blood lipid, coronary heart disease, inflammatory cytokines and cell adhesion molecules, microRNA‐34a, stenosis degree

## Abstract

**Background:**

MicroRNA‐34a (miR‐34a) plays an essential role in regulating blood lipid, inflammation, cell adhesion molecules, and atherosclerosis, the latter factors are closely involved in the etiology of coronary heart disease (CHD). However, the clinical value of miR‐34a in CHD patients' management is rarely reported. Hence, this study aimed to assess the correlation of miR‐34a with disease risk, blood lipid, coronary artery stenosis, inflammatory cytokines, and cell adhesion molecules of CHD.

**Methods:**

A total of 203 CHD patients and 100 controls were recruited in this study, then their plasma samples were collected to detect the miR‐34a by reverse transcription quantitative polymerase chain reaction. Furthermore, serum samples from CHD patients were obtained for inflammatory cytokines and cell adhesion molecule measurement by enzyme‐linked immunosorbent assay.

**Results:**

MiR‐34a was elevated in CHD patients compared to controls (*p* < 0.001) and it disclosed a good diagnostic value of CHD (area under curve: 0.899, 95% confidence interval: 0.865–0.934). Besides, miR‐34a positively correlated with triglyceride (*p* < 0.001), total cholesterol (*p* = 0.022) and low‐density lipoprotein cholesterol (*p* = 0.004), but not with high‐density lipoprotein cholesterol (*p* = 0.110) in CHD patients. Moreover, miR‐34a associated with Gensini score in CHD patients (*p* < 0.001). As to inflammation‐related indexes and cell adhesion molecules, MiR‐34a expression was positively linked with C‐reactive protein (*p* < 0.001), tumor necrosis factor alpha (*p* = 0.005), interleukin (IL)‐1β (*p* = 0.020), IL‐17A (*p* < 0.001), vascular cell adhesion molecule‐1 (*p* < 0.001), and intercellular adhesion molecule‐1 (*p* = 0.010) in CHD patients, but not with IL‐6 (*p* = 0.118) and IL‐10 (*p* = 0.054).

**Conclusion:**

MiR‐34a might serve as a biomarker in assistance of diagnosis and management of CHD.

## Graphical abstract

This study enrolled 203 CHD patients and 100 patients with unexplained chest pain or suspected CHD symptoms but excluded from CHD by CAG examination as controls, then plasma samples were collected to detect miR‐34a. Besides, for CHD patients only, serum samples were obtained for inflammatory cytokine and cell adhesion molecule measurement. Interestingly, miR‐34a was increased in CHD patients and disclosed a good diagnostic value of CHD. Moreover, miR‐34a positively correlated with hyperlipidemia, hyperuricemia, and DM in CHD patients. Furthermore, miR‐34a positively associated with blood lipid (including TG, TC and LDL‐C) and Gensini score in CHD patients. Additionally, miR‐34a positively related to inflammation‐related indexes (including CRP, TNF‐α, IL‐1β and IL‐17A) and cell adhesion molecules (including VCAM‐1 and ICAM‐1) in CHD patients.

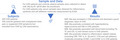

## 1 INTRODUCTION

Coronary heart disease (CHD), mainly caused by atherosclerotic plaques as well as narrowness and occlusion of artery lumen, remains to be the leading cause of cardiovascular mortality with nearly 360 thousand deaths in the United States in 2018.^1^, ^2^, ^3^, ^4^ Moreover, CHD is often associated with other complications such as hypertension, diabetes mellitus (DM), and hyperuricemia, which increases difficulties in the management of CHD.^5^, ^6^, ^7^ Although many treatments have been applied (including anti‐ischemic, antiplatelet therapy, lipid lowering treatment, coronary revascularization, and exercise treatment), the prognosis of patients is still unsatisfying and CHD continues to bring large burden to patients such as reduced quality of life and enormous medical costs.^8^, ^9^, ^10^ Hence, new markers are necessary to provide better risk stratification and more effective intervention for CHD patients.

MicroRNA‐34a (miR‐34a) locates on chromosome 1p36.23 and mostly expresses in adipocytes, macrophages, endothelial cells, smooth muscles, etc.^11^, ^12^, ^13^ Some studies indicate that miR‐34a plays essential roles in atherosclerosis (AS) by regulating macrophage cholesterol, blood lipid, inflammatory cytokines, and cell adhesion molecules, etc..^12^, ^14^, ^15^ For instance: (1) MiR‐34a causes increased triglyceride accumulation and attenuates lipid metabolism by inhibiting the Sirtuin 1 (SIRT1)‐related pathway.^16^ (2) MiR‐34a stimulates the secretion of pro‐inflammatory cytokines by triggering nuclear factor‐kappaB (NF‐κB) signaling pathway and downregulating SIRT1 activity.^17^ In addition, miR‐34a expands the generation of Th17 cells by repressing forkhead box protein 3 (FOXP3) expression.^18^ (3) MiR‐34a is able to induce vascular cell adhesion molecule‐1 (VCAM‐1) and intercellular adhesion molecule‐1 (ICAM‐1) protein expression and further aggravates endothelial cell inflammation.^19^ Additionally, increased blood lipid, proinflammatory cytokines, and highly expressed cell adhesion molecules are closely implicated in the etiology of CHD.^20^, ^21^, ^22^ Based on what was mentioned above, we assumed that miR‐34a associated with the pathogenesis of CHD and could serve as a biomarker for CHD management.

Hence, this study aimed to explore the correlation of miR‐34a with disease risk, blood lipid, coronary artery stenosis degree, inflammatory cytokines, and cell adhesion molecules of CHD.

## 2 METHODS

### 2.1 Study population

Between March 2018 and November 2020, a total of 203 CHD patients were continuously included in this study. The enrolled patients (with age ≥18 years old) were admitted to the hospital due to unexplained chest pain or suspected CHD symptoms, then they underwent coronary angiography (CAG) and were diagnosed as CHD according to at least one major epicardial vessel with >50% stenosis indicated by CAG.^23^ At the time of inclusion, patients with the following conditions were excluded: congenital heart diseases, inflammatory diseases, autoimmune diseases, active infections, hematological system diseases, tumors, bone marrow, or lymphatic system diseases. In addition to CHD patients, another 100 patients (with age ≥18 years old) with unexplained chest pain or suspected CHD symptoms who were admitted to the hospital but excluded from CHD by CAG examination were enrolled as controls, which included the patients with cardiac neurosis and the patients with microvascular angina (syndrome X). All controls were required to be age‐ and gender matched to CHD patients, without any of the following conditions: congenital heart diseases, inflammatory diseases, autoimmune diseases, active infections, hematological system diseases, tumors, bone marrow, or lymphatic system diseases. Besides, pregnant or lactating patients were not included in both CHD cohort and control cohort. The approval for the present study was obtained from the Institutional Review Board. All patients in this study signed the informed consents.

### 2.2 Data documenting

The clinical features of CHD patients and controls were documented after diagnostic workup, which mainly included demographic characteristics, comorbidities, smoke status, family history of CHD, and blood biochemical indexes. Those biochemical indexes were detected by Thermo Scientific™ Indiko™ (Catalog# 9863000, Waltham, Massachusetts, United States). Besides, the Gensini scoring system was applied for determining the severity of coronary artery stenosis,^24^ and the Gensini score of CHD patients and controls was also recorded for analysis. In detail, each lesion was assigned a score according to the percentage of stenosis: 1 for 25% stenosis, 2 for 50%, 4 for 75%, 8 for 90%, 16 for 99%, and 32 for total occlusion. Each principal vascular segment was assigned a coefficient: 5 points for left main coronary lesion; 2.5 points for proximal left anterior descending branch and left circumflex artery; 1.5 points for middle left descending artery lesion; 1 point for first diagonal branch and obtuse marginal branches and right coronary artery; 0.5 points for the second diagonal and posterolateral branch of the left circumflex artery. The direct stenosis score multiplied by the coefficient resulted in a final stenosis score of each segment, and the Gensini score of each patient was obtained by summing up the final stenosis score of each vascular segment.

### 2.3 Sample collection and processing

Whole blood samples of CHD patients were collected prior to CAG examination, which were divided into two parts: one was collected in a serum separator tube and left undisturbed at room temperature to clot for 15–30 min, then the clot was removed by centrifuging at 1000–2000 × *g* for 10 minutes in a refrigerated centrifuge, and the serum was immediately transferred into a clean polypropylene tube for detection; the other was collected in anticoagulant‐treated tubes and centrifuged twice at 1000–2000 × *g* for 10–15 min to remove cells and deplete platelets, then the resulting plasma was immediately transferred into a clean polypropylene tube for detection. In addition, the whole samples of controls were also extracted before CAG, which were collected in anticoagulant‐treated tubes and used for plasma collection as mentioned above.

### 2.4 Reverse transcription quantitative polymerase chain reaction assay

The miR‐34a expression in the plasma samples was determined by Reverse transcription quantitative polymerase chain reaction (RT‐qPCR) assay. Total RNA was extracted by QIAamp RNA Blood Mini Kit (Catalog# 52304, Qiagen), then reserve transcription was completed using QuantiTect Rev. Transcription Kit (Catalog# 205311, Qiagen). After that, qPCR was achieved by QuantiNova SYBR Green PCR Kit (Catalog# 208054, Qiagen). qPCR primers were designed referring to previous studies.^25^ The relative expression of miR‐34a was calculated by $2^{‐\Delta \Delta \text{CT}}$ method using U6 as the internal reference.

### 2.5 Enzyme‐linked immunosorbent assay

Enzyme‐linked immunosorbent assay (ELISA) was adopted to assess the levels of the inflammatory cytokines and the cell adhesion molecules in serum of CHD patients. Concretely, the inflammatory cytokines included tumor necrosis factor alpha (TNF‐α), interleukin (IL)‐1β, IL‐6, IL‐10, and IL‐17A; the cell adhesion molecules included VCAM‐1 and ICAM‐1. Commercial ELISA kits (Invitrogen, Carlsbad, California, USA) were applied for the assay, including TNF alpha Human ELISA Kit (Catalog# KAC1751), IL‐1 beta Human ELISA Kit (Catalog# KAC1211), IL‐6 Human ELISA Kit (Catalog# KAC1261), IL‐10 Human ELISA Kit (Catalog# KAC1321), IL‐17A Human ELISA Kit (Catalog# KAC1591), VCAM‐1 Human ELISA Kit (Catalog# KHT0601), and ICAM‐1 Human ELISA Kit (Catalog# BMS241). Assay procedures were implemented in strict accordance with experiment protocol provided by the manufacturer. The absorbance at 450 nm was read after the addition of stop solution immediately. A standard curve was performed with each assay, and the concentration of tested samples was read from the standard curve.

### 2.6 Statistical analysis

Features of variables were described using mean with standard deviation (SD), median with interquartile range (IQR) or count with percentage, as appropriate. Comparison between CHD patients and controls was checked by Student's *t* test, Mann‐Whitney *U* test, and Chi‐square test. The association between the two variables was analyzed by Spearman rank correlation test or Mann‐Whitney *U* test, as appropriate. The value of miR‐34a expression in distinguishing CHD patients from controls was evaluated by receiver operating characteristic (ROC) curve analysis. All tests were two sided, and a two‐side *p* value less than 0.05 indicated statistical significance. Data were analyzed by SPSS 26.0 (IBM Corp.), and figures were made by GraphPad Prism 7.01 software (GraphPad Software Inc.).

## 3 RESULTS

### 3.1 Characteristics of CHD patients and controls

The mean age of CHD patients and controls was 61.5±9.4 years and 62.0±6.7 years, respectively (*p* = 0.620, Table 1). CHD patients included 45 (22.2%) females and 158 (77.8%) males; meanwhile, 25 (25.0%) females and 75 (75.0%) males constituted the controls (*p* = 0.582). Notably, the differences of most clinical features were of no significance between CHD patients and controls, including body mass index (BMI), family history of CHD, comorbidities, fasting blood glucose (FBG), serum creatinine (Scr), serum uric acid (SUA), triglyceride (TG), high‐density lipoprotein cholesterol (HDL‐C) (all *p *> 0.050), except the proportion of smoking (*p* = 0.022), DM (*p* = 0.050), total cholesterol (TC) (*p* = 0.001), low‐density lipoprotein cholesterol (LDL‐C) (*p* < 0.001), and C‐reactive protein (CRP) (*p* = 0.010) (Table 1).

**TABLE 1:** Characteristics of CHD patients and controls

Items	Controls (N = 100)	CHD patients (N = 203)	Statistic (*t*/*χ^2^*/*Z*)	*p* value
Demographics				
Age (years), mean±SD	62.0 ± 6.7	61.5 ± 9.4	0.496	0.620
Sex, no. (%)				
Female	25 (25.0)	45 (22.2)	0.303	0.582
Male	75 (75.0)	158 (77.8)		
BMI (kg/m^2^), mean±SD	23.8 ± 3.1	24.2 ± 3.0	−1.169	0.243
Smoke, No. (%)				
No	68 (68.0)	110 (54.2)	5.274	0.022
Yes	32 (32.0)	93 (45.8)		
Family history of CHD, No. (%)				
No	79 (79.0)	152 (74.9)	0.629	0.428
Yes	21 (21.0)	51 (25.1)		
Comorbidities				
Hypertension, No. (%)				
No	28 (28.0)	49 (24.1)	0.527	0.468
Yes	72 (72.0)	154 (75.9)		
Hyperlipidemia, No. (%)				
No	57 (57.0)	95 (46.8)	2.789	0.095
Yes	43 (43.0)	108 (53.2)		
Hyperuricemia, No. (%)				
No	68 (68.0)	130 (64.0)	0.464	0.496
Yes	32 (32.0)	73 (36.0)		
Diabetes mellitus, No. (%)				
No	86 (86.0)	155 (76.4)	3.830	0.050
Yes	14 (14.0)	48 (23.6)		
Biochemical indexes				
FBG (mmol/L), median (IQR)	5.3 (4.8–6.0)	5.6 (4.8–6.3)	−1.248	0.212
Scr (μmol/L), median (IQR)	74.0 (64.4–86.4)	76.4 (67.5–85.3)	−1.125	0.260
SUA (μmol/L), median (IQR)	361.5 (315.8–414.8)	339.6 (301.4–396.0)	−1.580	0.114
TG (mmol/L), median (IQR)	1.4 (0.9–2.1)	1.7 (0.9–2.3)	−1.286	0.198
TC (mmol/L), mean±SD	4.3 ± 0.9	4.7 ± 1.1	−3.298	0.001
LDL‐C (mmol/L), median (IQR)	2.8 (2.3–3.3)	3.2 (2.5–4.0)	−4.227	<0.001
HDL‐C (mmol/L), mean±SD	1.0 ± 0.2	0.9 ± 0.2	1.419	0.157
CRP (mg/L), median (IQR)	6.5 (2.8–10.3)	7.4 (5.5–10.0)	−2.567	0.010
Stenosis degree				
Gensini score, mean±SD	1.1 ± 1.5	35.4 ± 23.0	−21.190	<0.001

Abbreviations: BMI, body mass index; CHD, coronary heart disease; CRP, C‐reactive protein; FBG, fasting blood glucose; HDL‐C, high‐density lipoprotein cholesterol; IQR, interquartile range; LDL‐C, low‐density lipoprotein cholesterol; Scr, serum creatinine; SD, standard deviation; SUA, serum uric acid; TC, total cholesterol; TG, triglyceride.

### 3.2 Comparison of miR‐34a expression between CHD patients and controls

**FIGURE 1:**
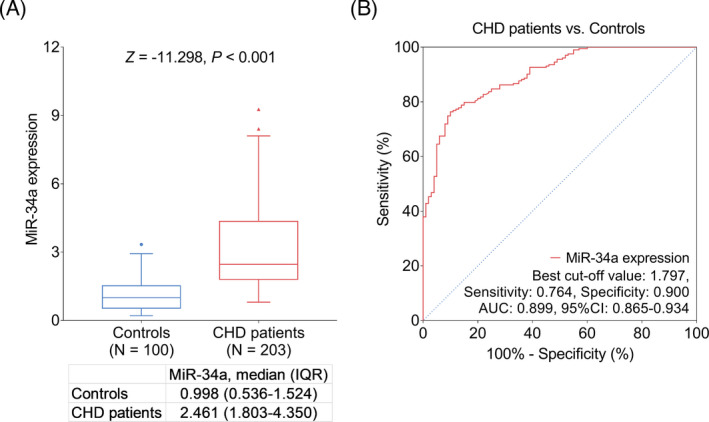
MiR‐34a expression in CHD patients and controls. Comparison of miR‐34a expression between CHD patients and controls (A) and diagnostic value of miR‐34a for CHD (B). CHD: coronary heart disease, miR‐34a: microRNA‐34a

MiR‐34a expression was elevated in CHD patients compared to controls (*p* < 0.001, Figure 1A). Furthermore, miR‐34a disclosed a good diagnostic value of CHD (area under curve (AUC): 0.899, 95% confidence interval (CI): 0.865–0.934), moreover, the sensitivity and specificity were 0.764 and 0.900, respectively, at the best cut‐off point (Figure 1B). Additionally, LDL‐C (AUC: 0.649, 95% CI: 0.588–0.710), TC (AUC: 0.597, 95% CI: 0.532–0.663), and CRP (AUC: 0.591, 95% CI: 0.515–0.666) had diagnostic values to distinguish CHD patients from controls, while FBG, Scr, SUA, TG, and HDL‐C failed to predict CHD risk (Figure S1A‐H).

### 3.3 Correlation of miR‐34a expression with the occurrence of comorbidities in CHD patients

There was no association between miR‐34a and the occurrence of hypertension (*p* = 0.949), while miR‐34A expression was positively correlated with the occurrence of hyperlipidemia (*p* = 0.022), hyperuricemia (*p* = 0.004), and DM (*p* < 0.001, Figure S2A‐D).

### 3.4 Correlation of miR‐34a expression with blood lipid and Gensini score in CHD patients

MiR‐34a expression was positively associated with TG (*r* = 0.314, *p* < 0.001, Table 2), TC (*r* = 0.161, *p* = 0.022), and LDL‐C (*r* = 0.202, *p* = 0.004), while miR‐34a expression was not correlated with HDL‐C (*r* = −0.113, *p* = 0.110).

**TABLE 2:** Correlation of miR‐34a expression with blood lipid level in CHD patients

Items	miR−34a expression	
	*r*	*p* value
TG	0.314	<0.001
TC	0.161	0.022
LDL‐C	0.202	0.004
HDL‐C	−0.113	0.110

Abbreviations: CHD, coronary heart disease; HDL‐C, high‐density lipoprotein cholesterol; LDL‐C, low‐density lipoprotein cholesterol; MiR‐34a, microRNA‐34a; TC, total cholesterol; TG, triglyceride.

**FIGURE 2:**
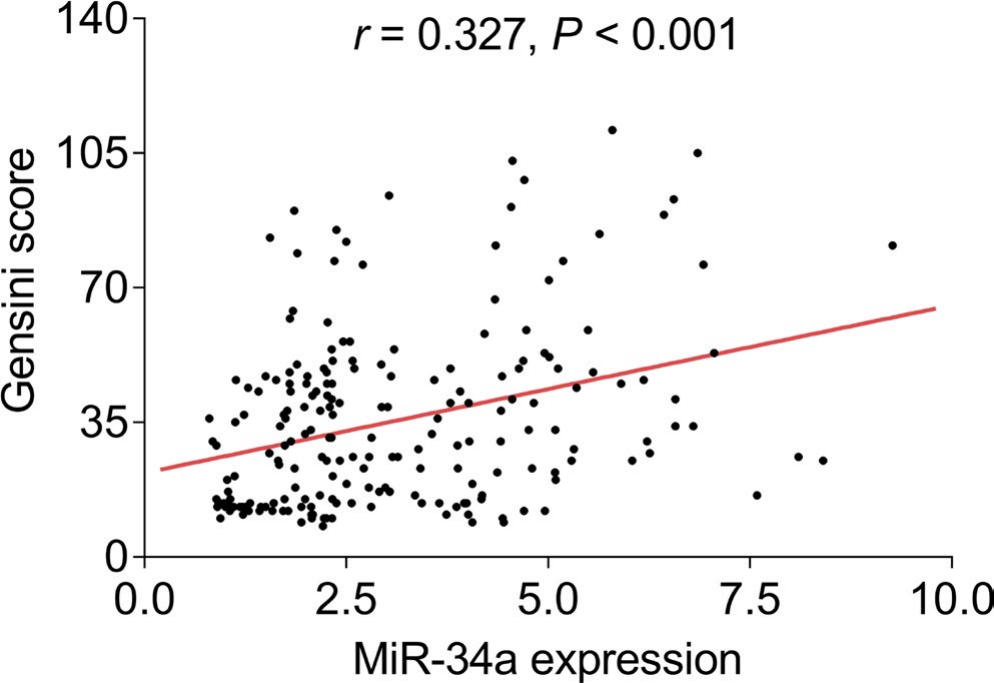
MiR‐34a was positively correlated with Gensini score in CHD patients. CHD: coronary heart disease, miR‐34a: microRNA‐34a

In terms of the relationship between miR‐34a and Gensini score, miR‐34a expression was positively correlated with Gensini score in CHD patients (*r* = 0.327, *p* < 0.001, Figure 2).

### 3.5 Correlation of miR‐34a expression with inflammatory cytokines and cell adhesion molecules in CHD patients

**FIGURE 3:**
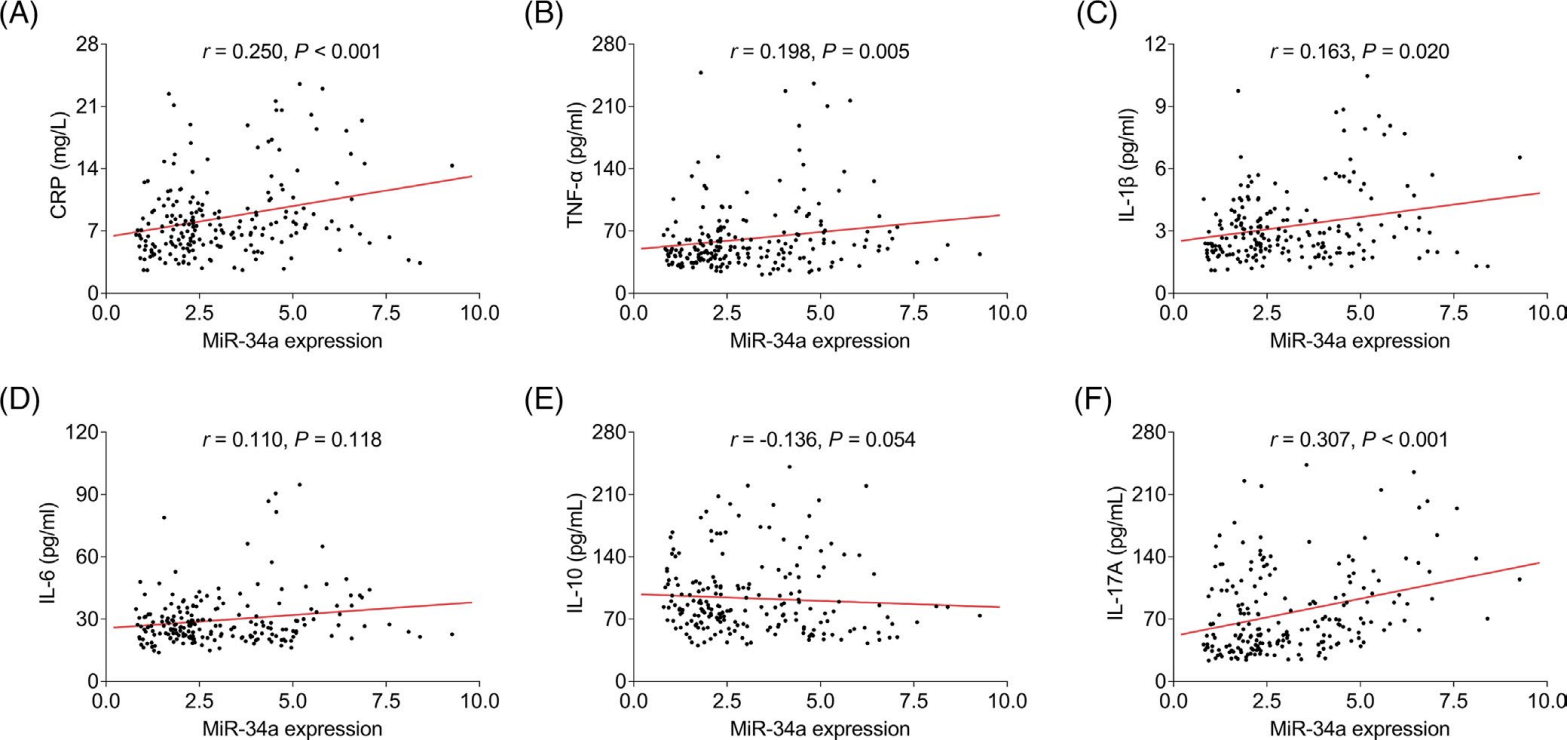
MiR‐34a was positively correlated with inflammation in CHD patients. Correlation of MiR‐34a with CRP (A), TNF‐α (B), IL‐1β (C), IL‐6 (D), IL‐10 (E), and IL‐17A (F) in CHD patients. CHD, coronary heart disease; miR‐34a, microRNA‐34a; CRP, C‐reactive protein; TNF‐α, tumor necrosis factor alpha; IL, interleukin

MiR‐34a expression was positively related to CRP (*r* = 0.250, *p* < 0.001), TNF‐α (*r* = 0.198, *p* = 0.005), IL‐1β (*r* = 0.163, *p* = 0.020), and IL‐17A (*r* = 0.307, *p* < 0.001) in CHD patients (Figure 3A‐C,F). Besides, no correlation of miR‐34a expression with IL‐6 (*p* = 0.118) and IL‐10 (*p* = 0.054) was observed (Figure 3D‐E).

**FIGURE 4:**
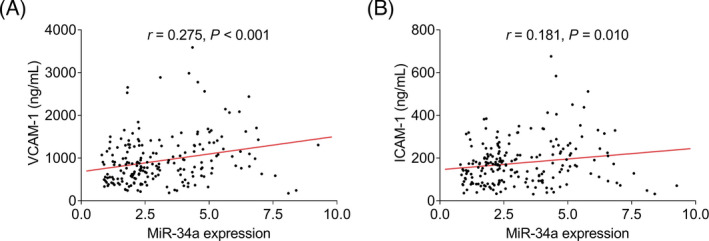
MiR‐34a was positively correlated with cell adhesion molecules in CHD patients. Correlation of MiR‐34a with VCAM‐1 (A) and ICAM‐1 (B) in CHD patients. CHD: coronary heart disease, miR‐34a: microRNA‐34a, VCAM‐1, vascular cell adhesion molecule‐1; ICAM‐1, intercellular adhesion molecule‐1

In terms of the correlation of miR‐34a with cell adhesion molecules, elevated miR‐34a expression was correlated with increased VCAM‐1 (*r* = 0.275, *p* < 0.001) and ICAM‐1 (*r* = 0.181, *p* = 0.010) in CHD patients (Figure 4A‐B).

### 3.6 Independent predictive factors of CHD risk

Multivariate logistic regression model was applied to further analyze the independent factors of CHD risk, which indicated that higher miR‐34a expression (odds ratio (OR): 8.035, 95% CI: 4.618–13.980, *p* < 0.001) and higher LDL‐C (OR: 2.332, 95% CI: 1.398–3.887, *p* = 0.001) were independently correlated with higher CHD risk, while higher HDL‐C (OR: 0.161, 95% CI: 0.033–0.793, *p* = 0.025) was independently linked with lower CHD risk (Table S1). Besides, we added the following formula for identifying the onset of CHD:

$$p=\frac{1}{1+\exp[‐(‐3.630+2.084*\text{miR}‐34a+0.847*\text{LDL}‐C‐1.827*\text{HDL}‐C)]}.$$

Also, we performed the ROC curve analysis to evaluate diagnostic value of the combination of three independent factors, which were found predict CHD risk (AUC: 0.912, 95% CI: 0.880–0.944, Figure S3).

## 4 DISCUSSION

This study mainly disclosed the following results: (1) MiR‐34a was increased in CHD patients than controls; meanwhile, it had a good diagnostic value of CHD. (2) MiR‐34a was positively correlated with blood lipid. (3) MiR‐34a was positively related to inflammatory cytokines and cell adhesion molecules. (4) Increased miR‐34a was associated with elevated coronary artery stenosis degree.

Accumulating lines of evidence find that miR‐34a is overexpressed in some cardiovascular diseases such as congenital heart disease, AS, and CHD.^14^, ^26^, ^27^ For instance, one study shows that miR‐34a was elevated in congenital heart disease patients than controls.^26^ Another study discloses that miR‐34a promotes aging of endothelial cells and is higher in the plasma of CHD patients than controls.^27^ Partially in line with previous studies, we found that miR‐34a expression was higher in CHD patients than in controls; meanwhile, miR‐34a could distinguish CHD patients from the controls. The possible reason could be that (1) MiR‐34a was known to be positively correlated with blood lipid and inflammatory cytokines, which were elevated in CHD patients.^14^, ^28^ Therefore, miR‐34a expression was higher in CHD patients than controls; (2) MiR‐34a inhibited the differentiation of vascular smooth muscle cells and promoted endothelial dysfunction by downregulation of Bcl2, which were positively related to the occurrence of CHD.^29^, ^30^, ^31^, ^32^ Therefore, miR‐34a could serve as a diagnostic biomarker for CHD patients. (3) MiR‐34a was known to be involved in atherosclerotic plaque development and lipid accumulation, the latter factors were pathologic changes of CHD; therefore, miR‐34a indicated a high risk of the development of CHD.^33^

Several studies also investigate miR‐34a expression in adipose tissues, which attributes to the acceleration of lipid accumulation and dyslipidemia in nonalcoholic fatty liver disease (NAFLD), type 1 diabetes mellitus (T1DM), etc.^16^, ^31^, ^34^, ^35^ Our study found that miR‐34a was positively associated with blood lipid in CHD patients. The possible explanation is as follows: (1) MiR‐34a directly reduced the expression of SIRT1 by binding to its 3′‐untranslated region (3’‐UTR), while SIRT1 was a key regulator in promoting lipid droplet catabolism.^16^, ^36^ Hence, miR‐34a was positively related to blood lipid in CHD patients. (2) miR‐34a attenuated metabolic action of FGF19 and FGF21 via downregulating β‐Klotho (βKL) expression; meanwhile, fibroblast growth factor (FGF)‐19 and FGF21 were metabolic hormones that accelerated lipid metabolism, increased miR‐34a was then correlated with elevated blood lipid.^37^

Apart from being closely related to blood lipid, miR‐34a also discloses a positive correlation with inflammatory factors in many diseases such as rheumatoid arthritis and endometritis.^18^, ^38^ For instance, one study finds that miR‐34a induces the release of the proinflammatory cytokines including IL‐1β, IL‐6, and TNF‐α in endometritis.^38^ However, no relevant study reports the association between miR‐34a and inflammation cytokines in CHD patients. Our study found that miR‐34a was positively correlated with inflammatory‐related indexes such as CRP, TNF‐α, IL‐1β, and IL‐17A. The probable explanations might be that: (1) SIRT1 attenuated the NF‐κB‐induced inflammatory response, while MiR‐34a downregulated SIRT1 activity.^17^ As a result, miR‐34a was positively correlated with inflammatory cytokines. (2) MiR‐34a induced the polarization of macrophages toward M1 and to secrete inflammatory cytokines, suggesting that miR‐34a might be related to an excessive inflammatory status.^39^ (3) MiR‐34a promoted the differentiation of CD4^+^ T cells into Th1 cells and Th17 cells, thus miR‐34a was positively correlated with their secreted inflammatory cytokines.^18^ Additionally, in our study, miR‐34a was also positively correlated with cell adhesion molecule (including VCAM‐1 and ICAM‐1). The possible reason might be that, as mentioned above, miR‐34a stimulated the NF‐κB signaling pathway while the NF‐κB pathway promoted the expression of cell adhesion molecules such as VCAM‐1 and ICAM‐1.^19^

Lastly, our study also showed that increased miR‐34a expression was related to elevated Gensini score in CHD patients. Possible reasons might be that the narrowness of artery lumen in CHD patients was mainly related to the emergence of atherosclerotic plaques, which would be aggravated due to lipid accumulation, increment of proinflammatory cytokines, and cell adhesion molecules.^40^ Additionally, as mentioned above, miR‐34a was positively correlated with blood lipid, inflammatory cytokines, and cell adhesion molecules; hence, miR‐34a expression was positively associated with Gensini score in CHD patients.

In terms of comorbidities of CHD patients, our study also found that elevated miR‐34a was linked with the occurrence of some comorbidities (including hyperlipidemia, hyperuricemia, and DM) in CHD patients. The probable reason might be as follows: MiR‐34a was known to regulate lipid metabolism via suppressing the expression of SIRT1; meanwhile, dyslipidemia was one of main factors causing these comorbidities.^30^, ^41^ Therefore, elevated miR‐34a was associated with some comorbidities in CHD patients.

Some limitations existed in the current study. Firstly, most patients in this study were elderly (mean age: 61.5 ± 9.4 years); therefore, the findings might not be suitable for younger CHD patients. Secondly, miR‐34a was detected at a single time point in this current study; therefore, its longitudinal change and value for continuously monitoring disease progression needed further exploration. Thirdly, the molecular mechanisms of miR‐34a on regulating the blood lipid remained controversial in a variety of diseases, which could be explored in CHD in further studies. Fourthly, fractional flow reserve was an important indicator to reflect the stenosis degree of CHD patients, which was necessary in further studies. Fifthly, the atherosclerotic plaque formation played crucial roles in the pathogenesis of CHD; thus, detailed relevant data were needed in the future studies. Sixthly, the correlation of miR‐34a with HDL‐C in CHD patients was relevantly weak, thus a multicenter study with larger sample size was required to further explore the issue.

Collectively, miR‐34a might serve as a biomarker in assistance of diagnosis and management of CHD.

## CONFLICTS OF INTEREST

The authors declare that they have no conflicts of interest.

## Supporting information

Fig S1

Fig S2

Fig S3

Table S1

## Data Availability

Data sharing not applicable to this article as no datasets were generated or analyzed during the current study.
