# Supplementary figures and images for: MicroRNA‐34a in coronary heart disease: Correlation with disease risk, blood lipid, stenosis degree, inflammatory cytokines, and cell adhesion molecules
Source: J Clin Lab Anal. 2021 Dec 3;36(1):e24138. doi: 10.1002/jcla.24138 (PMC8761464; doi:10.1002/jcla.24138)

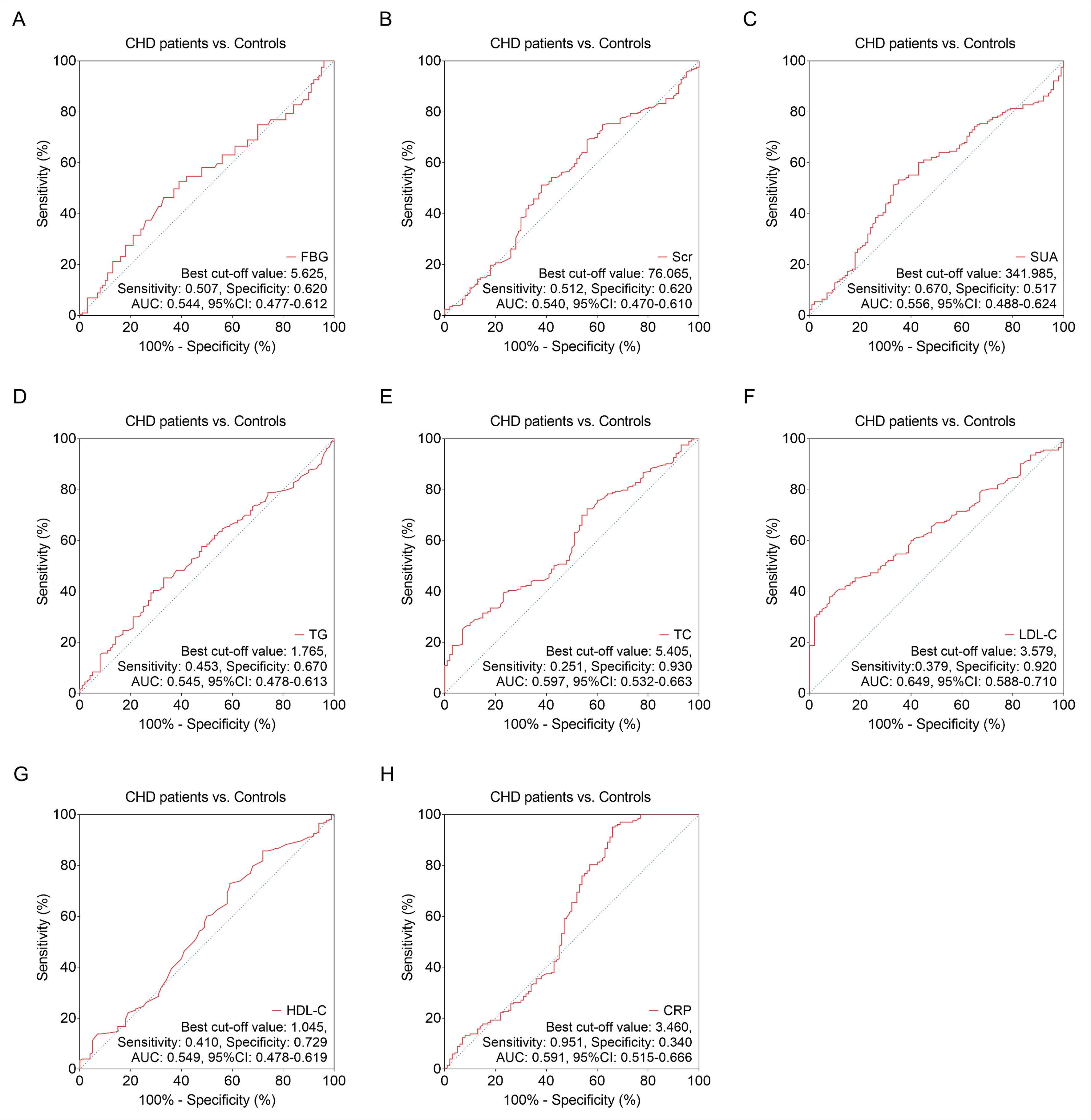

Supplement: Supplementary file 1 — Fig S1 [file JCLA-36-e24138-s004.tif]

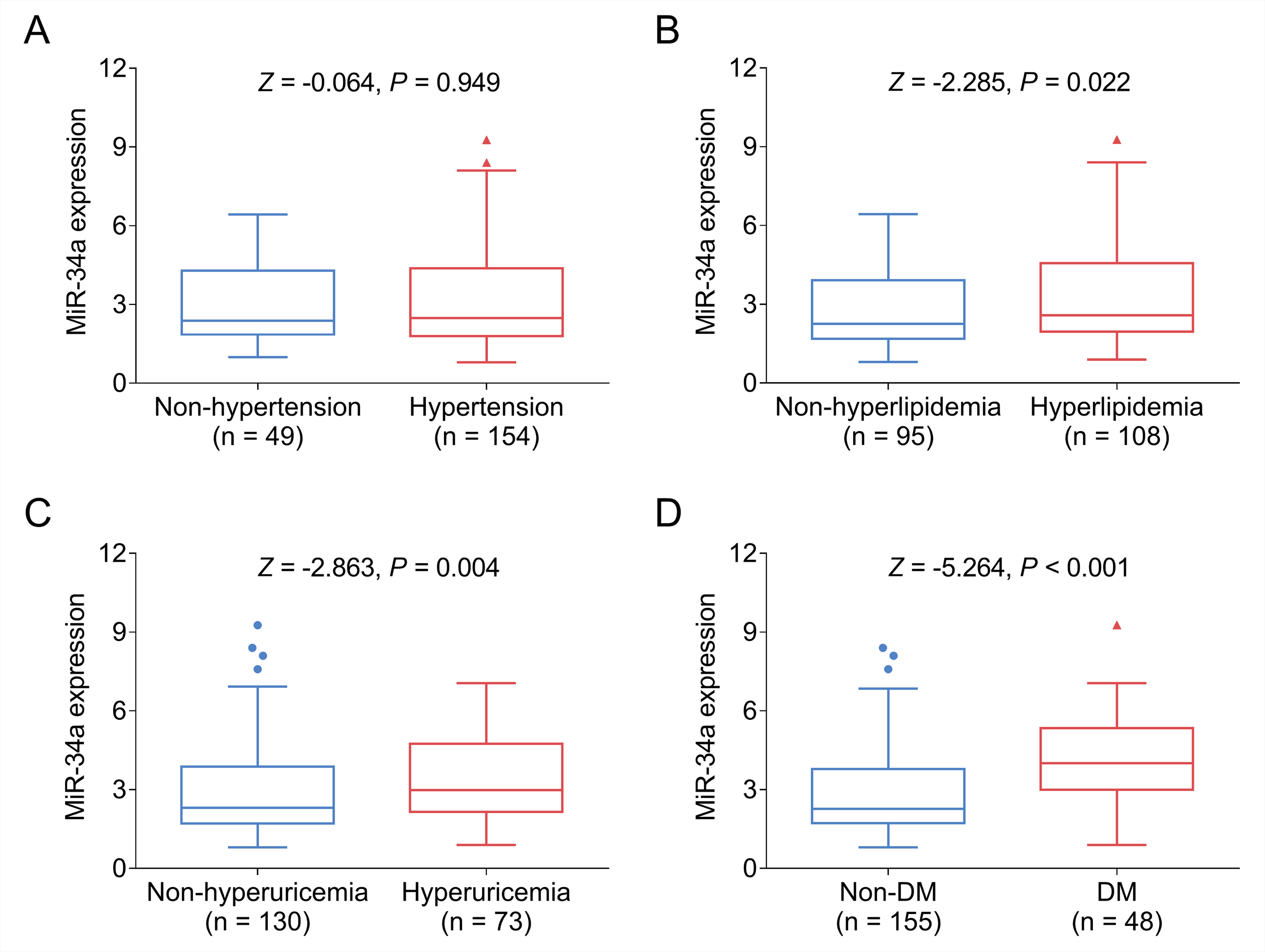

Supplement: Supplementary file 2 — Fig S2 [file JCLA-36-e24138-s002.tif]

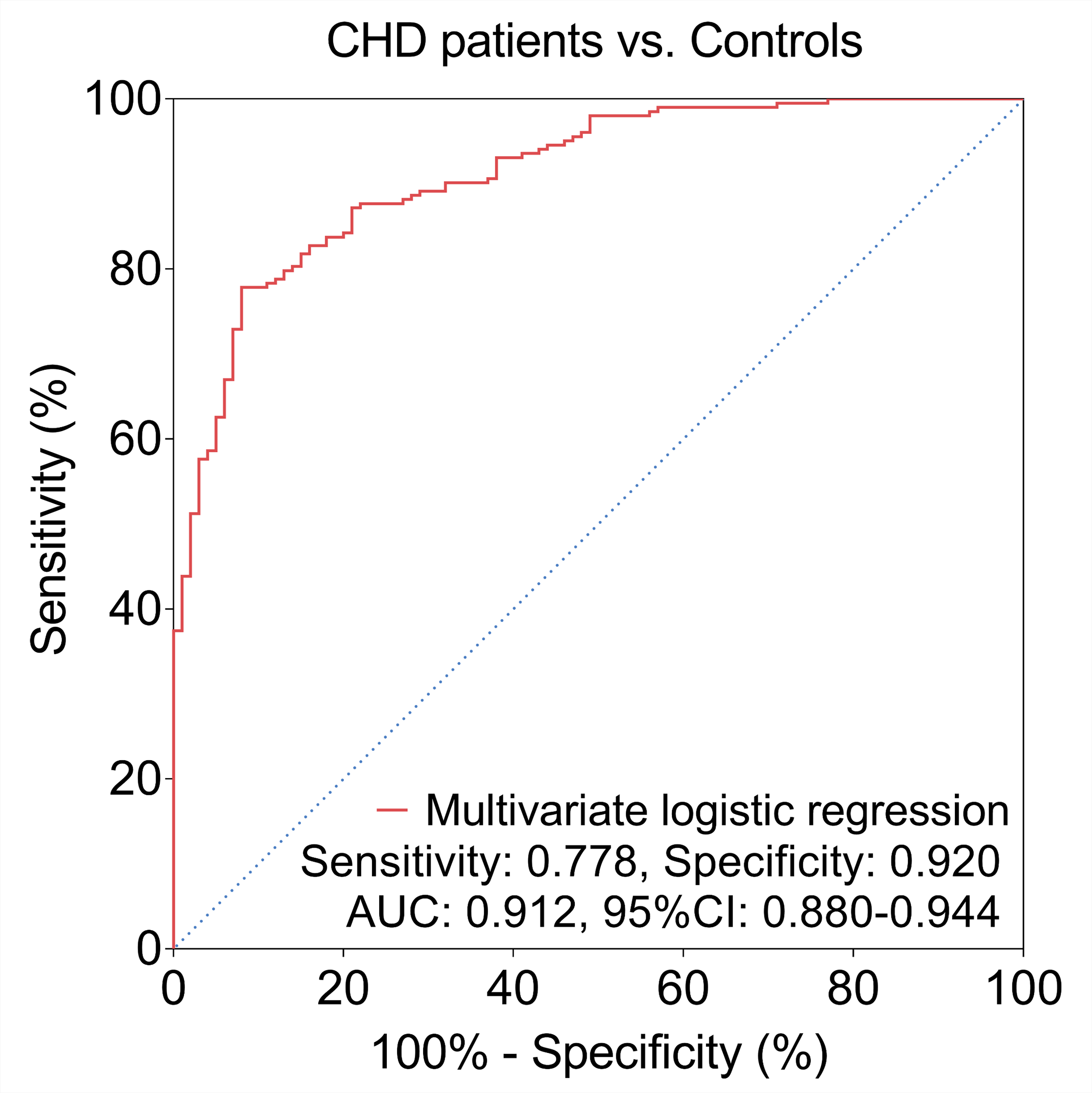

Supplement: Supplementary file 3 — Fig S3 [file JCLA-36-e24138-s001.tif]
