# Supplementary material for: MicroRNA‐34a in coronary heart disease: Correlation with disease risk, blood lipid, stenosis degree, inflammatory cytokines, and cell adhesion molecules
Source: J Clin Lab Anal. 2021 Dec 3;36(1):e24138. doi: 10.1002/jcla.24138 (PMC8761464; doi:10.1002/jcla.24138)
Supplement: Supplementary file 4 — Table S1 [file JCLA-36-e24138-s003.docx]

**Supplementary table 1.** Factors related to CHD risk by multivariate logistic regression model analysis

| Items | *P* value | OR | 95%CI | |
| --- | --- | --- | --- | --- |
|  |  |  | Lower | Upper |
| Higher miR-34a | <0.001 | 8.035 | 4.618 | 13.980 |
| Higher LDL-C | 0.001 | 2.332 | 1.398 | 3.887 |
| Higher HDL-C | 0.025 | 0.161 | 0.033 | 0.793 |

CHD, coronary heart disease; OR, odds ratio; CI, confidence interval; miR-34a, microRNA-34a; LDL-C, low-density lipoprotein cholesterol; HDL-C, high-density lipoprotein cholesterol. The regression model equation: $P=\frac{1}{1+exp[-(-3.630 + 2.084*miR-34a + 0.847*LDL-C -1.827*HDL-C)]}$.
